# SMZ/SNZ and gibberellin signaling are required for nitrate-elicited delay of flowering time in *Arabidopsis thaliana*

**DOI:** 10.1093/jxb/erx423

**Published:** 2017-12-22

**Authors:** Diana E Gras, Elena A Vidal, Soledad F Undurraga, Eleodoro Riveras, Sebastián Moreno, José Dominguez-Figueroa, David Alabadi, Miguel A Blázquez, Joaquín Medina, Rodrigo A Gutiérrez

**Affiliations:** 1 FONDAP Center for Genome Regulation, Millennium Nucleus Center for Plant Systems and Synthetic Biology, Departamento de Genética Molecular y Microbiología, Facultad de Ciencias Biológicas, Pontificia Universidad Católica de Chile, Santiago, Chile; 2 Instituto de Agrobiotecnología del Litoral (CONICET-UNL), Cátedra de Biología Celular y Molecular, Facultad de Bioquímica y Ciencias Biológicas, Universidad Nacional del Litoral, Paraje El Pozo, Santa Fe, Argentina; 3 Centro de Genómica y Bioinformática, Universidad Mayor, Santiago, Chile; 4 Centro de Biotecnología y Genómica de Plantas, (UPM-INIA) Campus de Montegancedo, Madrid, Spain; 5 Instituto de Biología Molecular y Celular de Plantas (CSIC-Universidad Politécnica de Valencia), Valencia, Spain

**Keywords:** Developmental transition, flowering, gibberellic acid, mineral nutrition, nitrate, nitrate transporter 1.1, Schlafmutze, Schnarchzapfen

## Abstract

The reproductive success of plants largely depends on the correct programming of developmental phase transitions, particularly the shift from vegetative to reproductive growth. The timing of this transition is finely regulated by the integration of an array of environmental and endogenous factors. Nitrogen is the mineral macronutrient that plants require in the largest amount, and as such its availability greatly impacts on many aspects of plant growth and development, including flowering time. We found that nitrate signaling interacts with the age-related and gibberellic acid pathways to control flowering time in *Arabidopsis thaliana*. We revealed that repressors of flowering time belonging to the AP2-type transcription factor family including *SCHLAFMUTZE* (*SMZ*) and *SCHNARCHZAPFEN* (*SNZ*) are important regulators of flowering time in response to nitrate. Our results support a model whereby nitrate activates SMZ and SNZ via the gibberellin pathway to repress flowering time in *Arabidopsis thaliana*.

## Teaser

Nitrate represses the gibberellin pathway and subsequently activates the flowering repressors SMZ and SNZ, delaying flowering time in Arabidopsis.

## Introduction

Nitrogen (N) is an essential component of many key biological molecules and a limiting factor for plant growth in natural as well as in agricultural systems (Frink *et al.*, 1999). N availability can have profound effects on a variety of developmental programs such as germination, seedling establishment, and flowering (Vidal *et al.*, 2014). Nitrate is one of the main sources of N in the soil. Changes in nitrate concentration are sensed by the transporter and receptor NITRATE TRANSPORTER 1.1 (NRT1.1) (Ho *et al.*, 2009). Nitrate perception is able to trigger signaling events that include an increase in cytoplasmic Ca^2+^, which acts as a second messenger (Gutiérrez, 2012; Riveras *et al.*, 2015). Nitrate signal transduction produces transcriptional changes in an extensive array of genes that play pivotal roles in N metabolism [e.g. nitrate transporters *NRT1.1*, *NRT2.1*, and *NRT2.2*, nitrate reductase (*NIR*), and nitrite reductase (*NIA1* and *NIA2*)] as well as in plant ontogeny [e.g. auxin receptor *AFB3*, bZIP transcription factors *TGA1* and *TGA4*, Arabidopsis Nitrate Regulated 1 (*ANR1*), lateral organ boundary domain (LBD37/38/39), among others] (Jonassen *et al.*, 2009; Rubin *et al.*, 2009; Gutiérrez, 2012; Alvarez *et al.*, 2014; Vidal *et al.*, 2014; O’Brien *et al.*, 2016).

Flowering is one of the most important developmental transitions during a plant’s life cycle (Koornneef *et al.*, 1998). Floral induction is regulated by an intricate genetic network that integrates both environmental and endogenous signals (Amasino, 2010; Fornara *et al.*, 2010; Srikanth and Schmid, 2011). Major environmental factors known to affect flowering time are the photoperiod (Imaizumi *et al.*, 2003; Imaizumi and Kay, 2006; Giakountis and Coupland, 2008; Michaels, 2009) and prolonged exposure to cold temperatures, a process known as vernalization (Alexandre and Hennig, 2008; Michaels, 2009). On the other hand, endogenous pathways include the gibberellic acid signaling pathway (Mutasa-Göttgens and Hedden, 2009), and the autonomous and age-related pathways that monitor plant developmental state (Simpson, 2004; Wang, 2014). These different pathways converge to regulate expression of a small number of flowering integrator genes that promote flowering, including *SUPPRESSOR OF OVEREXPRESSION OF CO1* (*SOC1*), *FLOWERING LOCUS T* (*FT*), and *LEAFY* (*LFY*) (Adrian *et al.*, 2009; Michaels, 2009; Amasino, 2010; Srikanth and Schmid, 2011).

It has been reported that *FT* encodes the ‘florigen’, a mobile signal that is produced in the leaf tissue and is transmitted to the shoot apical meristem, where it initiates flowering (Kobayashi *et al.*, 1999; Golembeski and Imaizumi, 2015). Due to its importance, *FT* is subjected to fine transcriptional control. Multiple transcriptional activators, such as GIGANTEA (GI) and CONSTANS (CO), bind to its promoter region; in contrast, repressors such as FLOWERING LOCUS C (FLC) and SHORT VEGETATIVE PHASE (SVP) down-regulate its expression (Sawa and Kay, 2011; Andrés and Coupland, 2012). SCHLAFMUTZE (SMZ) together with its paralog SCHNARCHZAPFEN (SNZ) are floral repressors from the *AP2* family of transcription factors. They delay flowering under long-day (LD) conditions and are targets of the microRNA miR172, a pivotal regulator of the ageing pathway. Chromatin immunoprecipitation experiments have demonstrated that SMZ is able to bind directly to the *FT* locus, down-regulating its expression (Mathieu *et al.*, 2009; Golembeski and Imaizumi, 2015).

Gibberellin (GA) is a plant hormone that regulates flowering time. When bioactive GAs bind to their receptors, they trigger the proteasome-dependent degradation of the DELLA transcription factors (Murase *et al.*, 2008; Shimada *et al.*, 2008; Mutasa-Göttgens and Hedden, 2009). These proteins regulate plant development and physiology by modifying the activity of a myriad of transcription factors, either by inhibiting their DNA binding ability or by acting as co-activators, facilitating their attachment to target promoters (de Lucas *et al.*, 2008; Feng *et al.*, 2008; Hou *et al.*, 2010; Zhang *et al.*, 2011; Hong *et al.*, 2012; Yang *et al.*, 2012; Xu *et al.*, 2014). Application of exogenous GAs promotes the transition from vegetative growth to flowering in a variety of plants (Bernier, 1988; Jacobsen and Olszewski, 1993; Chandler and Dean, 1994). The role of GAs in flowering initiation has been observed primarily in LD plants grown under non-inductive conditions. In Arabidopsis the photoperiodic pathway and its core components CO and FT dominate flowering initiation under inductive conditions (Mutasa-Göttgens and Hedden, 2009). Despite the dominance exerted by the CO-FT module under LD conditions, the GA biosynthesis mutant *ga1-3* and the triple GA receptor mutant *gid1* show a delayed flowering phenotype when grown under LDs, establishing a role for GAs under inductive conditions (Wilson *et al.*, 1992; Griffiths *et al.*, 2006; Mutasa-Göttgens and Hedden, 2009). Interestingly, there is evidence supporting an interaction between the GA pathway and nitrate nutrition. It has been shown that Arabidopsis plants grown under low-nitrate conditions have higher levels of bioactive GAs. It was proposed that low concentrations of nitrate activate the biosynthesis of GAs, as evidenced by increased expression of the GA biosynthetic enzyme GA1 under low nitrate (Liu *et al* 2013).

Nitrate and other N-nutrients or metabolites are known to modify flowering time in plants (Klebs, 1913; Dickens and Staden, 1988; Bernier *et al.*, 1993; Loeppky and Coulman, 2001). Arabidopsis plants grown under low-nitrate conditions flower earlier than plants grown under high nitrate (Castro Marín *et al.*, 2011; Yuan *et al.*, 2016). This effect was first attributed to a novel signaling pathway acting directly over floral integrators; however, the identity of the components involved in this pathway is still an open question (Castro Marín *et al.*, 2011; Kant *et al.*, 2011; Liu *et al.*, 2013). Yuan *et al.* (2016) found that N-signaling affects ferredoxin-NADP^+^-oxidoreductase (FNR1) and the blue-light receptor cryptochrome 1 (CRY1), causing a delay in flowering time. However, these studies used a mix of N nutrients and metabolites that did not narrow down the contribution of a specific N component to a particular signaling pathway.

In this work, we used molecular genetics approaches in order to find components involved in nitrate-dependent regulation of flowering time. We found that under N-sufficient conditions, nitrate delays flowering time by controlling the expression of the floral repressors *SMZ* and *SNZ*. Modulation of *SMZ* and *SNZ* gene expression by nitrate requires the GA pathway. Our results support a model whereby NRT1.1-mediated nitrate signaling interacts with the GA pathway and key elements of the ageing pathway in order to control bolting and flowering time in Arabidopsis.

## Materials and methods

### Plant material

Experiments were performed with *Arabidopsis thaliana* Columbia-0 (Col-0) and L*er* ecotypes as indicated. The following lines have been previously described: *chl1-5* (Liu *et al.*, 1999); *chl1-9* (Ho *et al.*, 2009); *toe1-2* and *toe2-1* (Aukerman and Sakai, 2003); *smz-2*, *snz-1*, *smz-2/snz-1*, and *toe1-2/toe2-1*, *smz-2/snz-1/toe1-2/toe2-1* (Mathieu *et al.*, 2009); *co* (SAIL24H04) (Kim and Michaels, 2006); *flc-3* (Michaels and Amasino, 1999); miR156 overexpressor (Schwab *et al.*, 2005); miR172 overexpressor (Mateos *et al.*, 2010); *rga-t2/gai-t6/rgl1-1/rgl2-1/rgl3-1* quintuple DELLA mutant (Feng *et al.*, 2008); *ft-10* (Yoo *et al.*, 2005); *soc1-2* (Lee *et al.*, 2000); the *RGA::GFP-RGA* line (Silverstone *et al.*, 2001); the overexpressor lines 35S::*GNL* (35S:*YFP:GNL*) and *35S::GNC* (35S:*GNC:GFP*), and the *gnc-gnl* double mutant (*gnc*, SALK_001778; *gnl*, SALK_003995) (Richter *et al.*, 2010, 2013*a*, 2013*b*).

### Growth and treatment conditions

Seeds were stratified at 4 °C for 3 d in complete darkness to synchronize germination, then sterilized and grown in plastic trays with vermiculite. Plants were watered with N-free medium (100 µM H_3_BO_3_, 3 mM CaCl_2_, 100 µM MnSO_4_, 0.16 µM CuSO_4_, 0.1 µM Na_2_MoO_4_, 1.25 mM KH_2_PO_4_, 1.5 mM MgSO_4_, 50 µM ZnSO_4_, 10 µM KI, 100 µM FeSO_4_, 100 µM Na_2_EDTA, and 0.1 µM CoCl_2_) supplemented with different concentrations of KNO_3_ as the only N source. A constant volume of nutrient solution per plant was applied once every week until flowering time. Flowering time was measured as the time between sowing and anthesis (opening of the first flower). Bolting time was recorded when the main inflorescence had reached a height of 0.5 cm. Plants were grown in a growth room, under a controlled environment, with a 16/8 h light/dark cycle, cool white fluorescent illumination of 100 μmol m^−2^ s^−1^ and a constant temperature of 22 °C. Leaf production rate was calculated as: number of leaves/days to bolting.

For gene expression assays, seeds were sown on vertical agar plates containing N-free medium supplemented with either 1 or 3 mM KNO_3_. Seedlings were grown for 7, 9, 11, 13, or 15 d on a Percival incubator (Percival Scientific, Inc.) under a 16/8 light/dark cycle at 22 °C. They were harvested at zeitgeber time 0 (ZT0) on these days.

### RNA quantification

Total RNA was isolated from seedlings with the mirVana kit (Life technologies, Carlsbad, CA, catalog no. AM1560) according to the manufacturer’s instructions. For mRNA quantification, reverse transcription was performed using the ImProm-II reverse transcriptase (Promega, Madison, WI). Quantitative real-time PCR was carried out in a StepOne Real time PCR system (Life technologies, Carlsbad, CA). The *ADAPTOR PROTEIN-4 MU-ADAPTIN* gene (At4g24550) was used as a housekeeping gene (Jonassen *et al.*, 2009; Rubin *et al.*, 2009). Quantification of miR172 levels was performed with the TaqMan microRNA ath-MIR172a assay (Life Technologies, Carlsbad, CA, catalog number 4427975). snoR41Y (Life Technologies, Carlsbad, CA, catalog number 4427975) was used as an internal reference. All experiments were carried out with three independent biological replicates.

### GFP-RGA imaging and quantification

Transgenic lines expressing *GFP* (green fluorescent protein) under the control of the *RGA* promoter (Silverstone *et al.*, 2001) were grown in N-free medium supplemented with either 1 or 3 mM KNO_3_ for 7 d. A Zeiss LSM780 confocal microscope was used for imaging. At least eight independent roots were photographed, and the number and relative intensities of GFP-fluorescent particles were automatically calculated using Fiji software (Schindelin *et al.*, 2012).

### Statistical analysis

Statistical analyses of bolting/flowering, rosette leaves, and gene expression data were done with one-way ANOVA and Tukey’s HSD tests using the GraphPad scientific software (Prism).

## Results

### Timing of bolting and flowering are regulated by nitrate in Arabidopsis

**Fig. 1.**
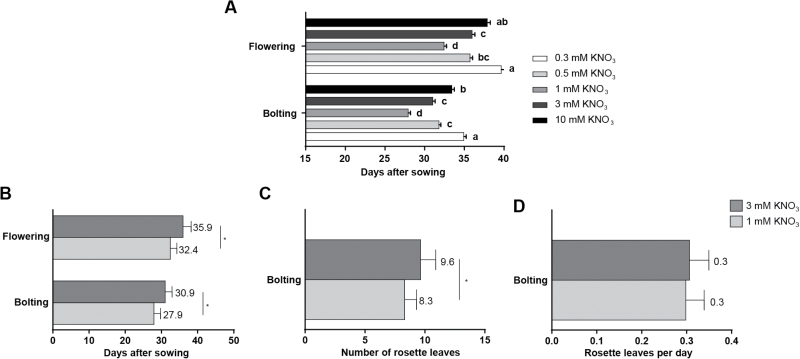
Flowering time varies according to nitrate availability. Arabidopsis plants were sown on vermiculite and watered once a week with N-free nutrient solution containing either 0.3, 0.5, 1, 3, or 10 mM KNO_3_. Number of days to bolting and flowering (A, B), number of rosette leaves at bolting (C), and average number of rosette leaves per day (D) were determined. At least 20 plants were used for each quantification. Data are means and SD of three independent biological replicates. Asterisks and different letters indicate significant differences as determined by Tukey’s Multiple Comparison test (*P*≤0.01).

To evaluate the effect of nitrate concentration on flowering time in Arabidopsis, we seeded plants on vermiculite and grew them under a long-day (LD) photoperiod at 22 °C constant temperature. Nitrate concentrations in agricultural soils typically average 6 mM (Crawford and Glass, 1998). Since the ion gets rapidly depleted from the soil solution, the most frequent concentrations to which plants are exposed oscillate between 2 and 5 mM (Crawford and Glass, 1998; Owen and Jones, 2001; Andrews *et al.*, 2013). Plants were fertilized weekly with a nutrient solution lacking nitrogen and supplemented with either 0.1, 0.3, 0.5, 1, 3, or 10 mM KNO_3_. (Fig. 1A). Plants grown with 0.1 mM KNO_3_ were unable to complete their life cycle under our experimental conditions. Plants grown with 0.3 or 0.5 mM KNO_3_ did complete their life cycle, but showed severe signs of N-limitation, including chlorotic leaves and reduced shoot development, as previously described (Bi *et al.*, 2007). Widely used parameters for assessing reproductive phase change and flowering time include recording the number of rosette leaves at bolting (floral stem at 0.1 cm height), or counting the number of days from sowing to bolting, or to flowering (Pouteau and Albertini, 2009). We found that an increased nitrate availability delayed both bolting and flowering time. Plants flowered faster at 1 mM and exhibited increasing delays at 3 mM or 10 mM nitrate concentrations. To further characterize the effect of nitrate on flowering time, we chose to do the rest of our experiments with 1 and 3 mM concentrations, to avoid the confounding effects of severe nutritional stress and to allow plants to complete their life cycle without N excess.

As shown in Fig. 1B, Arabidopsis plants grown with 3 mM KNO_3_ respectively bolted and flowered 3 and 3.5 d later than plants cultivated with 1 mM KNO_3_. In addition, plants grown at 3 mM KNO_3_ produced 1.3 more rosette leaves (Fig. 1C). These results are consistent with previous reports, which showed that increased nitrate concentrations delay flowering time as measured by number of rosette leaves or by number of days (Castro Marín *et al.*, 2011; Kant *et al.*, 2011; Liu *et al.*, 2013).

Interestingly, we found no differences in rosette leaf production rate between the two nitrate treatments (Fig. 1D). This suggests that nitrate availability has a direct impact on the phase change, but does not influence the normal rate of plant vegetative growth under our experimental conditions.

### Nitrate interacts with the gibberellin pathway and SMZ/SNZ floral repressors to control the reproductive phase transition in Arabidopsis

**Fig. 2.**
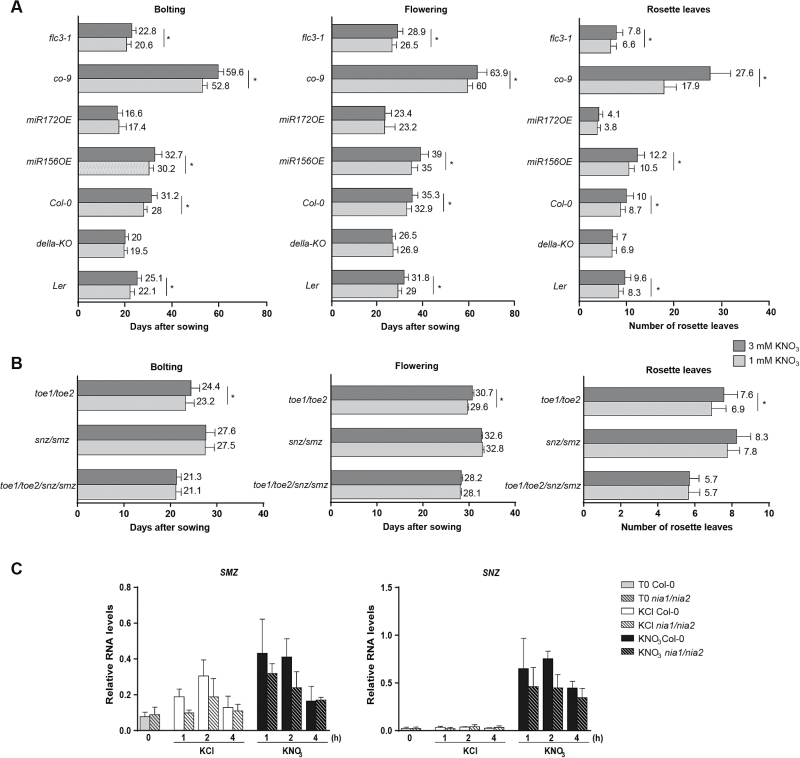
Nitrate-dependent delay in flowering time depends on the gibberellin pathway and the miR172 targets *SMZ* and *SNZ*. Plants were sown on vermiculite and watered once a week with N-free nutrient solution containing either 1 mM (light gray) or 3 mM (dark gray) KNO_3_. Nitrate-dependent flowering time and rosette leaf number of mutants for key genes from different flowering pathways were determined (A). Nitrate-dependent flowering and rosette leaf number of the quadruple *toe1/toe2/smz/snz* or double *toe1/toe2* and *smz/snz* mutants were also quantified (B). (C) Col-0 (filled bars) and nitrate reductase double-mutant (*nia1/nia2*) plants (dashed bars) were sown on a N-free hydroponic medium supplemented with 0.5 mM ammonium succinate and then treated with either 5 mM KCl or 5 mM KNO_3_ for the indicated times. Total RNA was extracted from shoots, and *SMZ* and *SNZ* transcript levels were quantified by qRT-PCR. The *ADAPTOR PROTEIN-4 MU-ADAPTIN* gene (*At4g24550*) was used as an internal reference. *flc3-1*, FLOWERING LOCUS C mutant; *co-9*, CONSTANS mutant; miR172OE, miR172 overexpressor; miR156OE, miR156 overexpressor; *della-KO*, quintuple DELLA mutant (L*er* background); *toe1, toe2*, TARGET OF EAT mutants; *snz*, SCHNARCHZAPFEN mutant; *smz*, SCHLAFMÜTZE mutant. All mutants and overexpressor lines, except *della-KO*, are in the Col-0 background. At least 20 plants were used for each flowering time measurement. Data are means and SD of three independent biological replicates. Asterisks highlight significant differences as determined by Tukey’s Multiple Comparison test (*P*≤0.01).

Plants have developed internal and environmentally dependent pathways to finely tune the timing of the reproductive phase transition according to endogenous and exogenous cues (Vidal *et al.*, 2014). In order to determine whether nitrate interacts with any of the previously described flowering pathways, we analysed different mutants on key genes for each pathway. We chose CONSTANS (CO), a central regulator of the photoperiodic pathway that stimulates flowering (Suárez-López *et al.*, 2001), Flowering Locus C (FLC), a strong flowering repressor from the autonomous and vernalization pathways (Koornneef *et al.*, 1994; Lee *et al.*, 2004), and a quintuple-DELLA mutant, *della-KO*, which lacks all five DELLA proteins and therefore shows constitutive, flowering-promoting GA signaling (Cao *et al.*, 2005). In addition, we evaluated plants that overexpress the miR156 and miR172 microRNAs, regulators of the age-dependent pathway (Wu *et al.*, 2009). We quantified the days to bolting and flowering as well as number of rosette leaves at bolting. We found that the repressive effect of nitrate over bolting and flowering was suppressed in the miR172 overexpressor and in the quintuple DELLA mutant (Fig. 2A). Interestingly, all the other mutants as well as the miR156 overexpressor showed the same bolting and flowering response to nitrate as the wild-type (Col-0) plants, with a delay at 3 mM KNO_3_ (Fig. 2A). These results indicate that nitrate interacts with the gibberellin pathway and with miR172 or its targets in order to regulate bolting and flowering time.

miR172 targets a set of AP2-like transcription factors that act as flowering repressors that down-regulate the floral integrator FT (Jones-Rhoades and Bartel, 2004; Mathieu *et al.*, 2009; Zhu and Helliwell, 2011). We found that a quadruple mutation of the AP2-like floral repressors *toe1*, *toe2*, *smz*, and *snz* abolished the repressive effect of nitrate over bolting and flowering (Fig. 2B). This effect was observed in *smz/snz* double- and single-mutants, but not in the *toe1 toe2* double-mutant (Fig. 2B, Supplementary Fig. S1 at *JXB* online). Consistently, we also found that both SMZ and SNZ were induced in nitrate-treated seedlings (Fig. 2C). Therefore, the effect of nitrate on flowering depends on the SMZ and SNZ floral repressors.

### Nitrate effect over bolting and flowering time depends on NPF6.3/NRT1.1

**Fig. 3.**
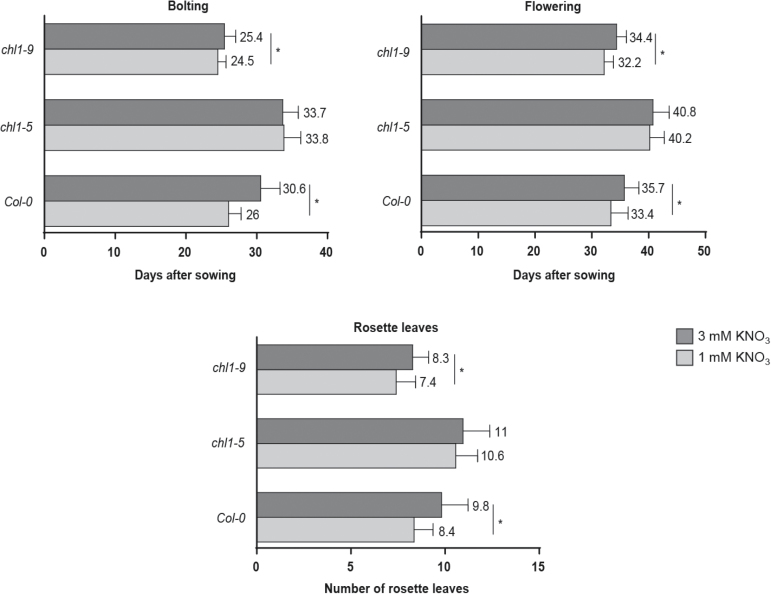
Nitrate repression of flowering is dependent on the NPF6.3/NRT1.1 nitrate transceptor. Nitrate transporter NPF6.3/NRT1.1 mutants *chl1-5* and *chl1-9* (Col-0 background) were sown on vermiculite and watered once a week with N-free nutrient solution containing either 1 mM (light gray) or 3 mM (dark gray) KNO_3_. At least 20 plants were used for each measurement. Data are means and SD of three independent biological replicates. Asterisks highlight significant differences as determined by Tukey’s Multiple Comparison test (*P*≤0.01).

The nitrate transporter and receptor NPF6.3/NRT1.1 is a key factor in the nitrate signaling pathway (Ho *et al.*, 2009; Bouguyon *et al.*, 2015; Riveras *et al.*, 2015). Mutant alleles of *NPF6.3/NRT1.1* are known to display a late-flowering phenotype when plants are grown on peat soil under LDs (Guo *et al.*, 2001). In order to determine whether this effect is nitrate-specific, we measured bolting and flowering in the loss-of-function *chl1-5* mutant (Liu *et al.*, 1999) grown in 1 and 3 mM nitrate. We found that bolting and flowering were delayed by 3 to 8 d in *chl1-5* mutant plants as compared to the wild-type (Figs 1 and 3). This delay was expected because the *chl1-5* mutant has impaired nitrate signaling and uptake (Liu *et al.*, 1999) and its flowering phenotype mimics wild-type plants growing under suboptimal nitrate concentrations (Fig. 1A). However, no differences in bolting or flowering times were observed between *chl1-5* mutant plants grown with 1 or 3 mM nitrate. This result indicates that NPF6.3/NRT1.1 is important for the effect of nitrate over the timing of the reproductive phase change and flowering. In order to determine whether nitrate repression of bolting and flowering depended on nitrate transport or a different function of NPF6.3/NRT1.1, we measured bolting and flowering in *chl1-9* mutant plants (Ho *et al.*, 2009). The *chl1-9* mutant has a P492L point-mutation that impacts specific aspects of NPF6.3/NRT1.1 function: it affects nitrate transport capacity in both the high-affinity and low-affinity range without an apparent effect on nitrate sensing and the downstream response of *NRT2.1* gene expression (Ho *et al.*, 2009; Bouguyon *et al.*, 2015). As shown in Fig. 3, *chl1-9* still had a nitrate-dependent flowering response. When grown with 3 mM KNO_3_, these plants bolted and flowered 0.9 and 2.2 d later, respectively, than their counterparts grown with 1 mM KNO_3_. These results indicate that nitrate-dependent repression of bolting and flowering is not dependent on nitrate transport by NPF6.3/NRT1.1 at the plasma membrane. Consistent with a signaling role of nitrate in controlling bolting and flowering, we found that nitrate was able to induce *SMZ* and *SNZ* gene expression in wild-type shoots as well as in the nitrate reductase double-mutant *nia1/nia2* (Wang *et al.*, 2007) (Fig. 2C).

These results indicate that nitrate is the signal that triggers bolting and flowering repression by controlling the transcript levels of the *SMZ* and *SNZ* genes.

### Nitrate availability controls the developmental expression of the *SMZ* and *SNZ* floral repressors and the *FT* floral integrator

**Fig. 4.**
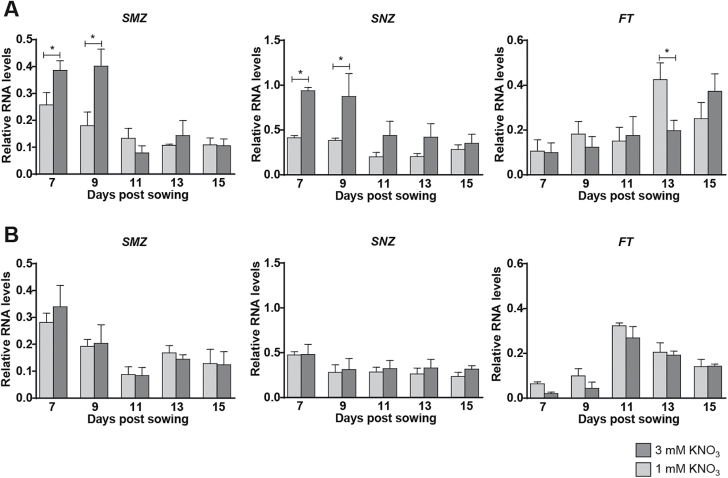
High nitrate availability induces a GA signaling-dependent early accumulation of *SMZ* and *SNZ* transcripts that correlates with a delayed increase in *FT* levels. Arabidopsis wild-type (A) and DELLA quintuple mutants (B) plants were grown on agar plates in N-free nutrient medium supplemented with either 1 mM (light gray) or 3 mM (dark gray) KNO_3_. At the days indicated, plants were harvested and RNA was extracted and used as a template for qRT-PCR. The *ADAPTOR PROTEIN-4 MU-ADAPTIN* gene (*At4g24550*) was used as an internal reference. Data are means and SE for three independent biological replicates of 15 plants. Asterisks highlight statistically different means as determined by Tukey’s Multiple Comparison test (*P*≤0.05).

Expression of floral repressors is developmentally regulated, being higher in young seedlings and gradually decreasing throughout the plant’s life cycle. Controlled expression of the floral repressors determines FT accumulation, which is crucial for determining the transition to flowering (Aukerman and Sakai, 2003). In order to determine whether nitrate controls bolting and flowering time by regulating expression of the *SMZ* and *SNZ* genes during development, we analysed the levels of these transcription factors over the first 2 weeks of Arabidopsis growth. We found that during early developmental stages, *SMZ* and *SNZ* gene expression levels were higher in plants grown with 3 mM KNO_3_. This difference disappeared on days 11–15, when they showed similar levels when compared to plants grown with 1 mM KNO_3_ (Fig. 4A). We found that the effect of nitrate concentration on SMZ and SNZ levels was not dependent on post-transcriptional regulation by miR172, since miR172 accumulates in plants grown under 1 mM KNO_3_ only at later time points (13 and 15 d post-sowing; Supplementary Fig. S2). Consistent with a repressor role of SMZ and SNZ over *FT*, mRNA levels of this floral integrator showed a peak of induction at day 13 only when plants were grown in 1 mM KNO_3_ (Fig. 4A).

**Fig. 5.**
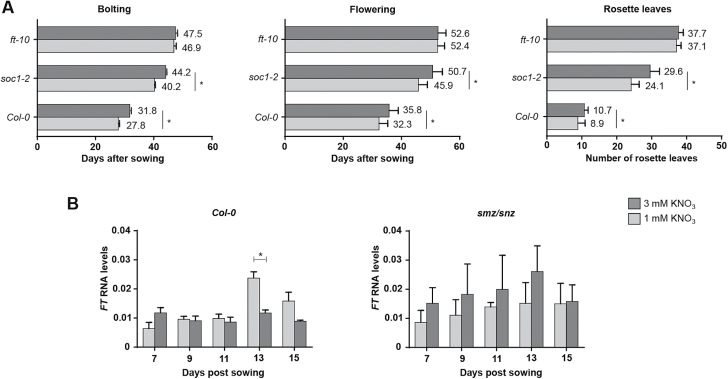
FT is the flowering integrator that mediates nitrate-dependent repression of flowering time. *ft-10* and *soc1-2* mutants (Col-0 background) were sown on vermiculite and watered once a week with N-free nutrient solution containing either 1 mM (light gray) or 3 mM (dark gray) KNO_3_. Nitrate-dependent flowering time and rosette leaf number were determined for 20–50 plants of each genotype (A). FT transcript levels of the wild-type (Col-0) and the *smz/snz* double-mutant (B). Plants were grown on Petri plates containing solid N-free nutrient medium supplemented with either 1 mM or 3 mM KNO_3_. At the days indicated, plants were harvested, their RNA was extracted and subsequently used for qRT-PCR. The *ADAPTOR PROTEIN-4 MU-ADAPTIN* gene (*At4g24550*) was used as an internal reference. Three independent biological replicates of 15 plants were used. Data are means and SD. Asterisks highlight significant differences as determined by Tukey’s Multiple Comparison test (*P*≤0.05).

It has been established that SMZ directly binds to the promoter of the floral integrators FT and SOC1 (Mathieu *et al.*, 2009). In order to further analyse the role of these integrators over nitrate-dependent flowering time, we tested flowering time of the loss-of-function mutants *soc1-2* and *ft-10*. As shown in Fig. 5A, flowering time of the *ft-10* mutant did not respond to different nitrate concentrations. On the contrary, *soc1-2* plants flowered later when grown under a higher KNO_3_ concentration. These results are consistent with a role for FT in nitrate-dependent control of flowering time in a SOC1-independent manner. Furthermore, quantitative RT-PCR showed that the FT transcript increase observed in 13-d-old seedlings grown under 1mM nitrate was suppressed in the *smz/snz* double-mutants (Fig. 5B). These data suggest that early accumulation of the floral repressors SMZ and SNZ under high nitrate leads to delayed bolting and flowering by controlling the expression of the *FT* floral integrator.

### Nitrate availability controls the developmental expression of GA biosynthesis genes and DELLA accumulation

Nitrate availability has been shown to control the levels of bioactive GAs to control flowering time (Liu *et al.*, 2013). In Arabidopsis, bioactive GA biosynthesis depends on oxidation of the GA precursors GA_12_ and GA_53_ by GIBBERELLIN-20-OXIDASE (GA20OX) into GA_9_ and GA_20_. This is followed by an oxidation step performed by GIBERELLIN-3-OXIDASE (GA3OX) family proteins to produce the bioactive forms GA_4_ and GA_1_ (Hedden and Phillips, 2000). We analysed the developmental expression of two members of these families, *GA20OX1* and *GA3OX1*, given their predominant role and expression in shoots (Rieu *et al.*, 2008). As shown in Supplementary Fig. S3, the expression of *GA20OX1* was lower in the early development of Arabidopsis when plants were grown in 3 mM as compared to 1 mM KNO_3_. We also found differences in the expression levels of *GA3OX1*, with an expression pattern under 1 and 3 mM KNO_3_ during development similar to the pattern of *FT* transcript accumulation (Figs 4A and 5B).

**Fig. 6.**
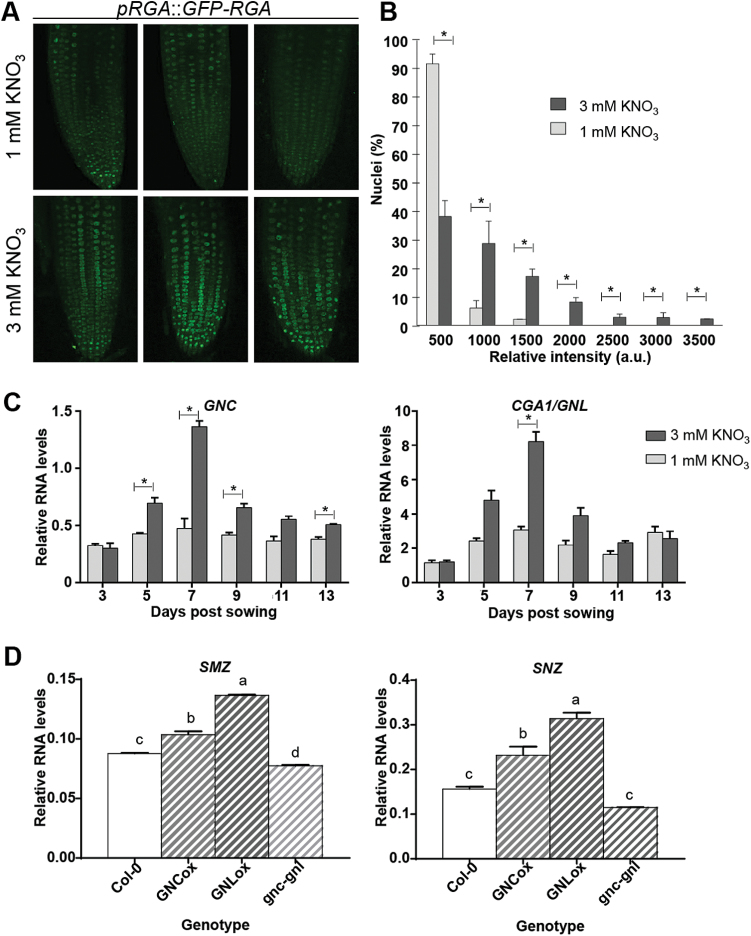
High nitrate availability promotes the accumulation of DELLA proteins and the induction of the downstream targets *GNC* and *CGA1/GNL*, triggering the up-regulation of *SMZ* and *SNZ* transcript levels. Representative confocal images showing roots of 7-d-old Arabidopsis *RGA::GFP-RGA* seedlings grown on agar plates in N-free nutrient medium supplemented with either 1 mM or 3 mM KNO_3_ (A). Quantification of the relative number of nuclei with specific GFP intensities (*n*=6) (B). Relative RNA levels for *GNC* and *CGA1/GNL* (C). Plants were grown on agar plates in N-free nutrient medium supplemented with either 1 mM (light gray) or 3 mM (dark gray) KNO_3_. At the days indicated, plants were harvested and RNA was extracted and used as a template for qRT-PCR. (D) Relative levels of *SMZ* and *SNZ*. Plants were grown on Petri plates containing solid N-free nutrient medium supplemented with 3 mM KNO_3_. On day 9, plants were harvested, their RNA was extracted and subsequently used for qRT-PCR. The *ADAPTOR PROTEIN-4 MU-ADAPTIN* gene (*At4g24550*) was used as a housekeeping gene. *GNC*ox, *GNC* overexpressor plants; *GNL*ox, *CGA/GNL* overexpressor plants; *gnc-gnl, gnc-cga/gnl* double-mutant plants. Data are means and SE for three independent biological replicates of 15 plants. Asterisks highlight significant differences as determined by Student’s *t*-test (*P*≤0.05). Different letters highlight significant differences as determined by Tukey’s Multiple Comparison test (*P* ≤0.05).

To confirm that nitrate-dependent differences in the expression levels of GA metabolism genes is biologically relevant, we analysed accumulation of the DELLA protein RGA in seedlings grown with 1 mM and 3 mM nitrate concentrations. DELLA proteins have been shown to accumulate when GA synthesis is impaired (Silverstone *et al.*, 2001; Stavang *et al.*, 2009). Consistently, we found that GFP-RGA levels were higher in roots of 7-d-old seedlings grown under 3 mM KNO_3_ (Fig. 6A, B), suggesting that high nitrate increases the levels of DELLA proteins by controlling the levels of bioactive gibberellins.

### Nitrate controls SMZ and SNZ levels in a GA-dependent manner

Our results are consistent with nitrate regulating the GA and age-related pathways to control bolting and flowering time. These pathways have been previously shown to interact to control flowering time (Porri *et al.*, 2012; Yu *et al.*, 2012; Hyun *et al.*, 2016). As previously shown in Fig. 2, the repressive effect of high nitrate concentration was abolished in the quintuple DELLA mutant, *della-KO* (Fig. 2A), similar to what was observed in the *smz/snz* mutant (Fig. 2B). In order to determine a possible crosstalk between the GA and the age-related pathways regarding nitrate-dependent bolting and flowering time, we analysed mRNA levels of the *SMZ* and *SNZ* genes in the *della-KO* mutant. As shown in Fig. 4B, mRNA levels of the *SMZ* and *SNZ* genes were similar when these mutant plants were grown in 1 or 3 mM KNO_3_. Moreover, *FT* mRNA levels peaked earlier and were similar in plants grown under either nitrate concentration, consistent with the bolting/flowering phenotype of the DELLA mutant. This suggests an interaction between DELLA proteins and SMZ/SNZ floral repressors to control FT levels depending on nitrate availability.

We also measured transcript accumulation of *GNC* and *CGA1/GNL*, which are transcription factors that act downstream of DELLA proteins, repressing certain GA responses (Richter *et al.*, 2010). Consistent with our previous findings, the expression levels of both genes were higher under 3 mM KNO_3_ than 1 mM KNO_3_ (Fig. 6C). We also found that GNC and CGA1/GNL affected *SMZ* and *SNZ* expression, as evidenced by changes in *SMZ* and *SNZ* expression in mutants and transgenic lines that had altered levels of *GNC* and/or *GNL* (Fig 6D). These results provide additional evidence for a role of GA signaling in the transcriptional control of *SMZ* and *SNZ*.

**Fig. 7.**
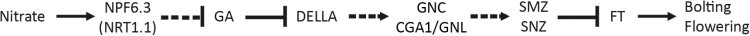
Model for regulation of flowering time by nitrate in *Arabidopsis thaliana*. A nitrate signal, sensed by the NPF6.3/NRT1.1 transceptor, represses a gibberellin signaling pathway that in time causes an early induction of *SMZ* and *SNZ*. Induction of *SMZ* and *SNZ* leads to early repression of the floral integrator *FT*, and a delay of flowering time. This model summarizes our results in a naive lineal model. However, it does not preclude multiple entries stemming from upstream signaling components (e.g. NRT1.1) over the effectors and, similarly, feedback regulation by effectors (e.g. GA pathway) towards upstream components. Dashed lines denote likely indirect regulation. The link between DELLA and the effectors GNC-CGA1/GNL is indirect and has been previously described (Richter *et al.*, 2010).

Our findings prompt a model whereby nitrate signaling controls bolting and flowering time by a GA-dependent pathway that leads to changes in the levels of *SMZ*, *SNZ*, and *FT* throughout the development of the plant (Fig. 7).

## Discussion

In order to ensure reproductive success, plants have evolved mechanisms to sense environmental and internal cues to tightly control the timing of the transition from vegetative to reproductive growth and their flowering time. Research in the last decade has led to the identification of major pathways controlling this transition, as well as a considerable array of its molecular components. Pathways controlling flowering converge in floral pathway integrators, including FT, which encodes the florigen, a flowering signal that migrates from the leaf to the shoot apex (Corbesier *et al.*, 2007; Mathieu *et al.*, 2007; Andrés and Coupland, 2012). In this work, we have shown that nitrate, the main N source available in agricultural soils, modified the timing of the reproductive phase change and flowering by interacting with two endogenous pathways controlling this process, the GA pathway and the age-related pathway.

Nitrogen is the mineral nutrient that is required in largest amounts by plants (Epstein and Bloom, 2005). Its availability has a direct effect over fitness, as plants grown with higher N concentrations produce higher seed yields (Araus *et al.*, 2016). Nitrate, the main N source for plants, triggers gene expression changes that encompass about 10% of the plant transcriptome. Nitrate-dependent gene networks are extraordinarily complex and have the ability to adjust in response to environmental perturbations. Nitrate assimilation integrates internal signals, such as carbon and energy metabolism, and environmental ones, such as light and N availability (Krouk *et al.*, 2010). It has long been known that N modifies flowering time in plants, with N limitation often inducing early flowering (Klebs, 1913; Dickens and Staden, 1988; Bernier *et al.*, 1993; Loeppky and Coulman, 2001). Other abiotic cues such as salt, drought, heat, cold, and UV radiation alter flowering time as well and this has been interpreted as a strategy that ensures seed production (Martínez *et al.*, 2004; Achard *et al.*, 2006).

Consistent with our findings, previous analyses of nitrate control of flowering have shown that nitrate availability modulates this developmental transition in Arabidopsis, with low nitrate availability accelerating and high nitrate availability delaying flowering time (Castro Marín *et al.*, 2011; Kant *et al.*, 2011; Liu *et al.*, 2013; Yuan *et al.*, 2016). However, these studies did not address whether nitrate interacts with the age-dependent pathway, limiting their results to analyses of the photoperiod, GA, and autonomous pathways (Castro Marín *et al.*, 2011; Kant *et al.*, 2011; Liu *et al.*, 2013). As we have shown in our work, nitrate regulation of *SMZ* and *SNZ* levels led to changes in the timing of *FT* expression, and consequently altered bolting and flowering time. During the plant’s life cycle, the levels of the *SMZ* and *SNZ* floral repressors peaked at early stages and diminished as plants aged, partially by the post-transcriptional control exerted by miR172 microRNA (Aukerman and Sakai, 2003). Our results show that nitrate availability was able to control early accumulation of *SMZ* and *SNZ* mRNAs. Overexpression of *SMZ* and *SNZ* has been shown to repress *FT* expression, causing a late-flowering phenotype under long-day conditions, with no effect under short days (Mathieu *et al.*, 2009). Accordingly, the timing of *FT* transcript accumulation was determined by nitrate availability under our experimental conditions, and was delayed under nitrate-sufficient conditions. Furthermore, the influence of nitrate over *FT* transcript accumulation was lost in the *smz/snz* double-mutant. Our data suggest that the nitrate-dependent control of *SMZ* and *SNZ* expression was mediated by a miR172-independent mechanism. The timing of nitrate-mediated changes in miR172 expression did not support an influence of this microRNA over the nitrate-elicited increase of *SMZ* and *SNZ* transcripts. In addition, the other tested targets of this microRNA, *TOE1* and *TOE2*, did not have roles in nitrate-dependent flowering time. Notwithstanding this, our results do not exclude a potential complementary role of miR172 at later time points or under different experimental conditions.

Regulation of *SMZ*, *SNZ*, and *FT* expression by nitrate was similar to what has been described for the control of flowering by trehalose-6-phosphate (T6P). This sugar directly controls transcript levels of *FT* and *SPL* family members in leaves, independently from miR156 (van Dijken *et al.*, 2004; Wahl *et al.*, 2013), contrasting with the miR156-dependent pathway, which is controlled by other sugars such as sucrose and glucose (Yang *et al.*, 2013; Yu *et al.*, 2013). Analysis of *SMZ* and *SNZ* gene expression in *della-KO* plants grown under low- and high-nitrate conditions indicated that nitrate controlled *SMZ* and *SNZ* mRNA levels by interacting with the GA pathway. A crosstalk between the GA pathway and nitrate-dependent flowering time had previously been suggested from transcript expression studies of genes involved in GA biosynthesis, floral meristem identity, and floral integrators (Kant *et al.*, 2011; Liu *et al.*, 2013). Consistent with a role for GA in controlling nitrate-regulated bolting and flowering, we found that the transcript levels of two key enzymes in active GA biosynthesis depended on nitrate availability. Transcriptomic data from multiple independent studies has also shown down-regulation of the GA biosynthetic genes *GA2*, *GA3OX1*, and *GA3OX4* in response to nitrate treatments (Canales *et al.*, 2014). Nitrate availability has been shown to control the levels of bioactive GA_3_, with low and high nitrate causing an increase and a reduction in its levels, respectively, by controlling the expression of *GA1*, a key enzyme in GA biosynthesis in Arabidopsis (Liu *et al.*, 2013). In agreement, our analysis of downstream effectors from the GA pathway showed that they were also affected by nitrate. First, the DELLA protein RGA accumulated more in the presence of a higher nitrate concentration. Second, the transcript levels of the downstream targets *GNC* and *CGA1/GNL* were up-regulated under these conditions. Both results showed that the GA pathway was more active at 1 mM than at 3 mM nitrate, consistent with the flowering time phenotypes observed under these conditions.

The GA and age-dependent flowering pathways are integrated by direct physical interaction between DELLA and SPL proteins (Porri *et al.*, 2012; Yu *et al.*, 2012; Hyun *et al.*, 2016). This interaction down-regulates the SPL-dependent transcription of *miR172* and MADS box genes. (Yu *et al.*, 2012). miR172 targets AP2-like flowering repressors, including *SMZ* and *SNZ* (Mathieu *et al.*, 2009). We found nitrate-dependent changes in *SMZ* and *SNZ* levels, which would suggest a GA-dependent down-regulation through the DELLA-SPL-miR172 module. However, our data also showed that nitrate-dependent flowering time was not affected in plants overexpressing miR156, which targets SPLs. These results strongly suggested that, for nitrate-dependent flowering time, the gibberellin pathway regulates *SMZ* and *SNZ* expression levels directly and that this is sufficient to explain the phenotype. Indeed, we obtained evidence identifying a cross-regulatory point between the two pathways. As shown in Fig. 6D, we found that two downstream targets of the GA pathway, *GNC* and *CGA/GNA*, had an effect over the expression of the flowering repressors *SMZ* and *SNZ*. This evidence was obtained with two transgenic lines overexpressing either *GNC* or *CGA/GNA*, and with a *gnc-cga/gna* double-mutant. Although both overexpressors consistently showed increased *SMZ* and *SNZ* levels, the double-mutant only showed a significant decrease of *SMZ* transcripts. This suggests that additional factors in the GA pathway may also regulate *SMZ/SNZ* expression.

Castro Marín *et al.* (2011) reported that nitrate regulates floral induction in Arabidopsis, acting independently of light, GA, and the autonomous pathways. The discrepancy between their conclusions regarding the role of GAs and ours could be attributed to multiple factors. First, their use of a combination of inorganic and organic N sources (nitrate and glutamine) with higher concentrations as compared to our study, where we focused on the effect of nitrate alone at relatively low concentrations. It has been shown that these different N sources can trigger disparate phenotypical effects (Zhang *et al.*, 1999; Alboresi *et al.*, 2005). Second, Castro Marin *et al.* (2011) tested flowering-time mutants in the gibberellin pathway under a neutral (12 h day/12h night) photoperiod and not LD conditions (16 h day/8 h night). Yuan *et al.* (2016) reported that N-dependent changes in flowering time are caused by variations in transcript levels of ferredoxin-NADP^+^-oxidoreductase (FNR1) and the blue-light receptor cryptochrome 1 (CRY1). The experimental conditions used in this study differed from ours in two key aspects. First, the N source used in this study was Murashige and Skoog medium supplemented with different concentrations of nitrate and ammonia. Second, flowering time was assessed in sterile Petri dishes under tissue-culture conditions, an environment that significantly differed from our set-up. These differing experimental settings may explain our disparate conclusions. This evidence, together with the Castro Marin results, strongly suggests that different N metabolites have effects over different pathways in order to control flowering time.

In Arabidopsis, nitrate availability is sensed by the NPF6.3/NRT1.1 nitrate transceptor (Ho *et al.*, 2009). Besides its role as a major nitrate uptake transporter in Arabidopsis roots, NPF6.3/NRT1.1 has been shown to have diverse signaling mechanisms independent of nitrate transport (Bouguyon *et al.*, 2015). According to our results, nitrate regulation of flowering time depended on a signaling function of nitrate that was dependent on NPF6.3/NRT1.1 since we did not find alterations in flowering time control in a NPF6.3/NRT1.1 mutant that was only altered in its nitrate uptake capability (Ho *et al.*, 2009; Bouguyon *et al.*, 2015). As for flowering, the control of seed dormancy by nitrate is also dependent on the NPF6.3/NRT1.1 transceptor (Alboresi *et al.*, 2005), suggesting that signaling by NPF6.3/NRT1.1 might represent a mechanism to coordinate developmental transitions to optimal environmental conditions. A potential connection between NPF6.3/NRT1.1 and the gibberellin pathway is supported by a microarray analysis that was performed with the NPF6.3/NRT1.1 loss-of-function allele *nrg1* and its wild-type counterpart (Wang *et al.*, 2009). When treated with 1 mM KNO_3_, *nrg1* mutants showed a 2-fold reduction of the gibberellin biosynthetic enzyme GA3OX1 (At1g15550) and the DELLA target CP1 (At4g36880), suggesting a role for NPF6.3/NRT1.1 in nitrate-dependent regulation of gibberellin (Cao *et al.*, 2006; Wang *et al.*, 2009). Furthermore, the complex relationship between N nutrition and the GA pathway is highlighted by a recent report that showed that the nitrate/nitrite transporter NPF3, a member of the same gene family of the *NPF6.3/NRT1.1* transceptor, is a GA transporter (Tal *et al.*, 2016).

Our data are consistent with a model in which nitrate availability controls GA activity, leading to an early regulation of *SMZ* and *SNZ* expression levels, which in turn changes the timing of *FT* induction and bolting and flowering time.

In summary, our phenotypical characterization together with our molecular genetics approach has uncovered a novel mechanism for nitrate-dependent control of flowering time. The shift from vegetative to reproductive development is one of the most important transitions throughout a plant’s ontogeny and, consequently, it is tightly controlled by various genetic and environmental factors. Although many environmental factors such as light, photoperiod, and temperature have been identified and characterized, the influence of mineral nutrients over flowering has been under-explored. Therefore, uncovering the molecular mechanisms of control that N availability exerts over the regulation of flowering time highlights the importance of nutritional status with regards to developmental decisions. Furthermore, it also provides new targets for crop improvement in a key environmental factor that impacts on reproductive success and yield.

## Supplementary data

Supplementary data are available at *JXB* online.

Fig. S1. Nitrate-dependent delay in flowering time is suppressed in the *smz* and *snz* single-mutants.

Fig. S2. miR172 expression is affected by nitrate availability at later stages of plant development.

Fig. S3. Nitrate availability controls the levels of active gibberellin key biosynthetic enzymes.

Supplementary Figures S1-S3

## Abbreviations:

GA: Gibberelic acid
NRT1.1: nitrate transporter 1.1
SMZ: Schlafmutze
SNZ: Schnarchzapfen.
