# Supplementary material for: SMZ/SNZ and gibberellin signaling are required for nitrate-elicited delay of flowering time in Arabidopsis thaliana
Source: J Exp Bot. 2017 Dec 22;69(3):619–31. doi: 10.1093/jxb/erx423 (PMC5853263; doi:10.1093/jxb/erx423)
Supplement: Supplementary Figures S1-S3 [file erx423_suppl_supplementary_figures_s1-s3.pdf]

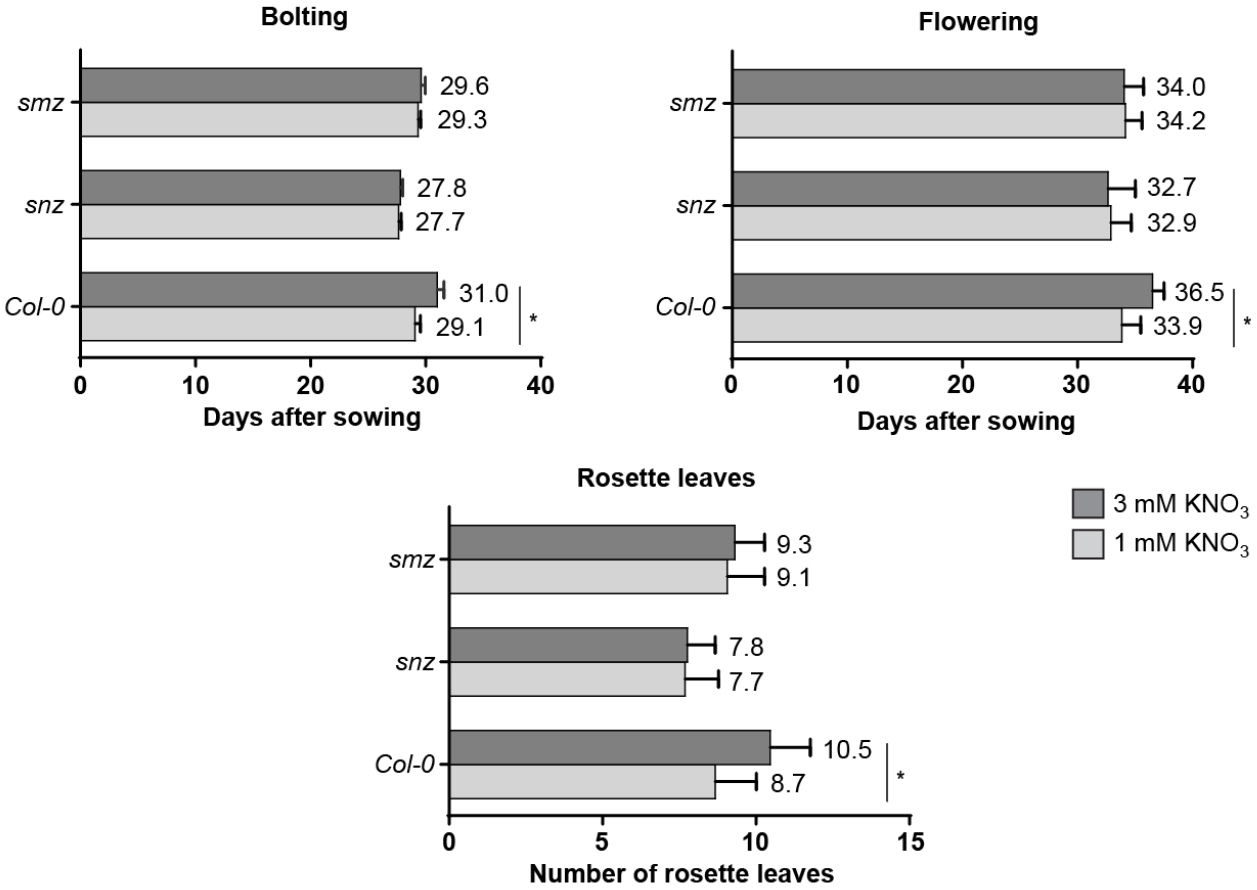

Figure S1. Nitrate-dependent flowering time delay is suppressed in *smz* and *snz* single mutants. Plants were sown on vermiculite and watered once a week with an N-free nutrient solution containing either 1 mM (light gray) or 3 mM (dark gray) KNO_3. Nitrate-dependent flowering time of the *smz-2* and *snz-1* mutants was determined. 45 plants were used for each measurement. Mean and standard deviation are shown. Asterisks highlight significant differences by Tukey's Multiple Comparison test (p-value ≤ 0.05).

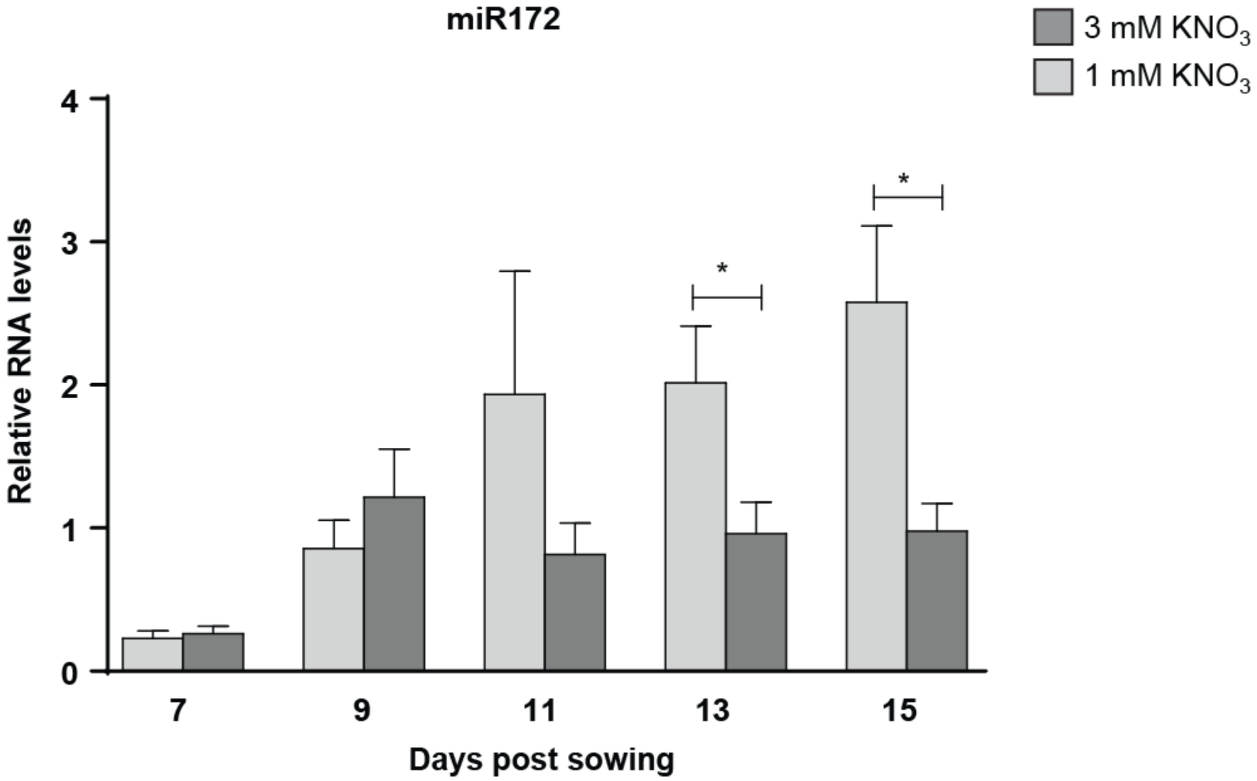

Figure S2. miR172 expression is affected by nitrate availability at later stages of plant development. Arabidopsis plants were grown on agar plates in N-free nutrient medium supplemented with either 1 mM (light gray) or 3 mM (dark gray) KNO_3. At the indicated days, plants were harvested and RNA was extracted and used as template for qRT-PCR using a TaqMan probe for miR172a,b. The snoR41Y gene (At5g66567) was used as internal reference. Mean and standard error for 3 independent biological replicates of 15 plants are shown. Asterisks highlight statistically different means by Tukey's Multiple Comparison test ($p \leq 0.05$).

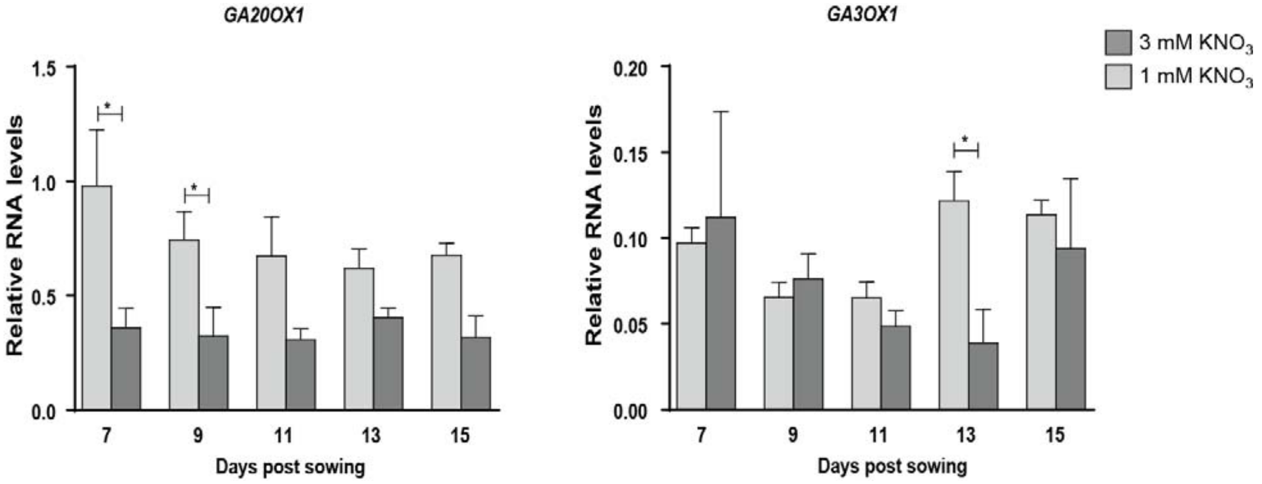

Figure S3. Nitrate availability controls the levels of active gibberellin key biosynthetic enzymes. Arabidopsis plants were grown on agar plates in N-free nutrient medium supplemented with either 1 mM (light gray) or 3 mM (dark gray) KNO_3. On the indicated days, plants were harvested and RNA was extracted and used as template for qRT-PCR. The *ADAPTOR PROTEIN-4 MU-ADAPTIN* gene (At4g24550) was used as internal reference. Mean and standard error for 3 independent biological replicates of 15 plants are shown. Asterisks highlight statistically different means by Tukey's Multiple Comparison test ($p \leq 0.05$).
